# Increased FSHD region gene1 expression reduces *in vitro* cell migration, invasion, and angiogenesis, *ex vivo* supported by reduced expression in tumors

**DOI:** 10.1042/BSR20171062

**Published:** 2017-10-27

**Authors:** Ankit Tiwari, Niharika Pattnaik, Archita Mohanty Jaiswal, Manjusha Dixit

**Affiliations:** 1 School of Biological Sciences, National Institute of Science Education and Research, HBNI, PO: Bhimpur-Padanpur, Via: Jatni, Khurda 752050, Odisha, India; 2 Department of Pathology, SRL Diagnostics Ltd, Plot 2084, Hall Plot 339/4820, Goutam Nagar Unit no. 28, Bhubaneswar, Khurda 751014, Odisha, India

**Keywords:** angiogenesis, cell migration, cell invasion, FRG1, Immunohistochemistry, oncogenesis

## Abstract

Facioscapulohumeral muscular dystrophy (FSHD) region gene 1 (FRG1) is a candidate gene for FSHD. FRG1 regulates various muscle-related functions, but studies have proposed its role in development and angiogenesis also, where it is involved with tumor-associated molecules. Therefore, we decided to look into its role in tumor progression, tumor angiogenesis, and its impact on cellular properties. Cell proliferation, migration, invasion and *in vitro* angiogenesis assays were performed to decipher the effect of FRG1 on endothelial and epithelial cell functions. Q-RT PCR was done for human embyonic kidney (HEK293T) cells with altered FRG1 levels to identify associated molecules. Further, immunohistochemistry was done to identify FRG1 expression levels in various cancers and its association with tumor angiogenesis. Subsequently, inference was drawn from Oncomine and Kaplan–Meier plotter analysis, for FRG1 expression in different cancers. Ectopic expression of FRG1 affected cell migration and invasion in both HEK293T and human umbilical vein endothelial cells (HUVECs). In HUVECs, FRG1 overexpression led to reduced angiogenesis *in vitro*. No effect was observed in cell proliferation in both the cell types. Q-RT PCR data revealed reduction in granulocyte-colony stimulating factor (G-CSF) expression with FRG1 overexpression and increased expression of matrix metalloproteinase 10 (MMP10) with FRG1 knockdown. Immunohistochemistry analysis showed reduced FRG1 levels in tumors which were supported by *in silico* analysis data. These findings suggest that reduction in FRG1 expression in gastric, colon and oral cavity tumor might have a role in tumor progression, by regulating cell migration and invasiveness. To elucidate a better understanding of molecular signaling involving FRG1 in angiogenesis regulation, further study is required.

## Introduction

Facioscapulohumeral muscular dystrophy (FSHD) region gene 1 (FRG1) is highly conserved from invertebrates to vertebrates [1]. Its exact function is unknown. FRG1 has been a candidate gene for FSHD [1]. Ectopic expression of FRG1 causes abnormal splicing of specific genes in mice, which leads to the development of FSHD-like phenotype [2]. Majority of studies have proposed a role of FRG1 in muscle development [1–5]. FRG1 overexpression leads to a reduction in myoblast cell proliferation, suggesting muscular atrophy [6]. Reduction in *frg1* levels in *Xenopus laevis* leads to disrupted muscle organization. Further, overexpression of *frg1* also leads to abnormal epaxial and hypaxial muscle development [3]. Altered expression of FRG1 not only affects muscle, but also the vasculature of the organism [7]. Vascular abnormalities have been observed in 75% of FSHD patients [8–10]. Reduction in *frg1* levels in *Xenopus laevis* reduced the levels of vascular marker *dab2* and vice versa [7]. Apart from above-mentioned studies, isolated studies are available about its role at a cellular level. Subcellular localization studies revealed that FRG1 is an actin bundling protein and localized in nucleolus or spliceosome complex, suggesting its role in RNA biogenesis [11,12]. Ectopically expressed FRG1 is localized in nuclear region, predominantly into nucleolus, Cajal bodies, and transcriptionally active chromatin regions [12,13]. Nonetheless, FRG1’s accurate function remains uncertain.

Various studies have indirectly associated FSHD with cancer. Treatment of dental epithelial cell line, mDEC6 with bone morphogenetic protein 4 (BMP4), a known tumor inhibitor, leads to translocation of FRG1 from the nucleus to the cytoplasm [14]. Moreover, FRG1’s functional domain analysis revealed that it consists of a fascin-like domain, a lipocalin domain, and two nuclear localization signals [11]. Fascins are actin bundling proteins which are crucial for tumor progression [15–18]. Transcriptional signature of FSHD myotubes and myocytes resemble highly with Ewing’s sarcoma [19]. Patients of muscular dystrophy such as Duchene muscular dystrophy (DMD), are known to develop cancer [20–23]. In terms of FSHD, there has been a single case report where FSHD patient was diagnosed with breast cancer [24].

Until now, there is no direct evidence showing the role of FRG1 in tumor angiogenesis and tumor progression. Similarly, the effect of FRG1 expression on other cell types, apart from myocytes and other muscle cells, is unknown. Above-mentioned studies suggest the role of FRG1 in development of various organs and angiogenesis. Therefore, FRG1 expression levels might be crucial for tumor progression through tumor angiogenesis or independently. The present study was taken up to explore the association of FRG1 expression with tumor progression. Further, to identify if this association is tumor angiogenesis dependent, we evaluated the effect of FRG1 expression in endothelial cells and epithelial cells.

## Materials and methods

### Plasmids, cell culture, and transfection

FRG1 expression vector (pCMV6.XL5.FRG1) and knockdown vector (pLKO.1. FRG1sh) along with their controls were procured from OriGene and Sigma, respectively. Plasmids were purified using Plasmid Mini Kit (Qiagen) using manufacturer’s guidelines. Human embyonic kidney (HEK293T) cells were obtained from National Center for Cell Science (NCCS), Pune, India and were grown in DMEM (PAN-Biotech) with 10% FBS (PAN-Biotech). HEK293T transfection was carried out using X-tremeGENE 9 (Roche) as per manufacturer’s protocol. Human umbilical vein endothelial cells (HUVECs) were procured from HiMedia Laboratories, Mumbai. HUVECs were maintained in HiEndoXL Endothelial Cell Growth Medium (HiMedia).

### Western blot

Cells were washed with ice-cold PBS and lysed in ice-cold radioimmunoprecipitation assay lysis buffer (Pierce), with added protease inhibitor (Sigma). In cell lysate, protein quantitation was done using BCA reagent (Pierce), as per manufacturer’s instructions. Thirty micrograms of protein sample was taken in fresh tubes and an equal volume of 2× Laemilli buffer was added to the cell lysate and boiled at 95°C for 10 min. The lysates were separated on SDS/PAGE (10% gel). The proteins on the gels were transferred oo PVDF membrane (Millipore). Then the blots were probed with an antibody specific to FRG1 (Novus Biologicals), and anti-mouse IgG secondary antibody (Pierce). The labeled bands were subsequently detected by chemiluminescence. For each sample, band intensities were normalized to GAPDH (Sigma) and β-tubulin (Cell Signaling Technology).

### Matrigel tubule formation assay

Matrigel (Corning) was thawed overnight in a 4°C refrigerator and subsequently plated 50 µl in a 96-well plate. Cell suspension was prepared using 0.5 × 10^4^ cells/ml in conditioned medium, obtained from HEK293T cells transfected with FRG1 expression vector and empty vector control. One hundred microliters of cell suspension was added to each well of Matrigel-coated 96-well plate and incubated at 37°C with 5% CO_2_. Images were taken after 6 h of incubation at 40× magnification in an inverted microscope (Ziess) and analysis was done using angiogenesis analyzer plugin in NIH ImageJ software.

### Cell proliferation assay

HEK293T cells (0.1 × 10^3^) were seeded in a 96-well plate. Cells were transfected with FRG1 expression and silencing vectors, along their respective controls, using Xtremegene9. Transfected cells were grown for 96 h. Afterward, the medium was replaced with fresh medium and 20 µl of MTS reagent (Promega). Absorbance was recorded at 490 nm wavelength using iMark Microplate Reader (Bio–Rad).

HUVECs (0.5 × 10^3^) were seeded in a 96-well plate in HiEndoXL Endothelial Cell Growth Medium (HiMedia). Post 24 h of seeding, endothelial cell growth medium was replaced with conditioned medium obtained from HEK293T cells transfected with FRG1 expression vector and respective vector control. HUVECs were grown in the respective conditioned medium for 96 h, followed by addition of fresh medium and 20 µl of MTS reagent (Promega).

### Wound healing assay

HEK293T cells (0.2 × 10^6^) were seeded in six-well plates. Transfection was done with FRG1 expression and silencing vectors, along with their respective controls using Xtremegene9. Cells were allowed to grow, till 100% confluence was achieved. Scratch was made in the plate using P200 pipette tip. Images were collected at 0 and 24 h under an inverted microscope (Ziess). Cell migration was analyzed using NIH ImageJ software. The experiment was conducted in triplicate.

### Transwell invasion and migration assay

Matrigel invasion assay was performed using Millipore Transwell chambers (8 μm: pore size); 2 × 10^4^ HEK293T cells were seeded in the upper chamber of a 12-well plate, coated with growth factor reduced Matrigel (Corning), in 500 μl serum-free medium. The lower chamber was filled with 500 μl medium containing 15% FBS to induce cell migration. The chamber was incubated at 37°C, for 24 h. At the end of incubation, cells in the upper surface of the membrane were removed with a cotton swab. Migrated cells, to the lower surface of the membrane, were fixed with methanol (Merck) and stained with Giemsa (HiMedia). The images were obtained using a CKX41 inverted microscope (Olympus) and the cells were counted in five different view fields, using NIH ImageJ software. The experiment was conducted in triplicate.

Transwell migration assay for HEK293T was performed with similar protocol as invasion assay except the transwell chambers were not coated with Matrigel. To understand the effect of FRG1 on endothelial cell migration, transwell migration assay for endothelial cells was performed in a co-culture setup. In this co-culture setup, HEK293T (epithelial cells) were cultured in the well of a plate and HUVECs (endothelial cells) were cultured in one chamber of Millipore Transwell chambers, which was kept in the same well. Thereafter, migration of endothelial cells to other side of transwell chamber, was studied. This setup could help us to identify the cross-talk between epithelial cells with altered FRG1 expression, and endothelial cells. HEK293T cells (0.1 × 10^6^) were seeded in a 12-well plate. HEK293T cells were transfected with FRG1 expression vector and empty vector control. After 48 h of HEK293T transfection, 2 × 10^4^ HUVECs were seeded in Millipore Transwell chambers (8 μm: pore size) which were placed in 12-well plates containing HEK293T cells. Post 24 h of incubation, HUVECs in transwell chamber were fixed with methanol and stained with Giemsa. Further analysis of migration was done as mentioned above.

### Quantitative real-time PCR

Total RNA was isolated with RNeasy Mini Kit (Qiagen) from HEK293T cells transfected with FRG1 expression vector and control vector, according to the manufacturer’s protocol. Total RNA concentration was measured using Nano Drop 2000 spectrophotometer (Thermo Fisher Scientific). RNA was converted into cDNA using the Superscript IV Reverse Transcriptase (Invitrogen). List of primers used for qRT-PCR for 7 matrix metalloproteinases (MMPs) and 11 tumor-associated cytokines, is given in the table (Supplementary Table S1). qRT-PCR was performed, using the Fast Start Universal SYBR Green Master Mix (Roche), in ABI 7500 system (Applied Biosystems). The experiment was conducted independently in three biological replicates, and GAPDH was used as an internal control.

**Table 1:** Data from tubule formation assay showing a change in various parameters of angiogenesis

S.No.	Angiogenesis analyzer parameter	FRG1 overexpression vector	Control vector	*P-*value
1	Number of extremities	132 ± 25	107 ± 14	0.026
2	Number of nodes	263 ± 98	371 ± 96	0.039
3	Number of junctions	79 ± 28	110 ± 28	0.041
4	Number of master junctions	32 ± 16	46 ± 15	0.073
5	Number of master segments	51 ± 29	80 ± 28	0.057
6	Total master segment length	6288 ± 2837	9571 ± 2576	0.028
7	Number of meshes	16 ± 12	27 ± 12	0.057
8	Total mesh area	127029 ± 120136	306016 ± 163722	0.022
9	Number of pieces	183 ± 33	218 ± 36	0.052
10	Number of segments	83 ± 41	130 ± 43	0.039
11	Number of branches	69 ± 7	70 ± 8	0.41
12	Number of isolated segments	30 ± 11	17 ± 8	0.025
13	Total length	13624 ± 1677	15602 ± 1613	0.029
14	Total branching length	11200 ± 2393	14257 ± 2200	0.029
15	Total segment length	5964 ± 2756	9165 ± 2562	0.029
16	Total branch length	5236 ± 683	5091 ± 777	0.36
17	Total isolated branches length	2424 ± 867	1345 ± 620	0.014
18	Branching interval	87 ± 43	132 ± 41	0.046
19	Mesh index	200 ± 26	212 ± 28	0.22
20	Mean mesh size	6830 ± 1740	10358 ± 3523	0.024

### Immunohistochemistry

Study included a total of 38 cases, containing squamous cell carcinoma of oral cavity (*n*=18), colorectal carcinoma (*n*=11), and gastric carcinoma (*n*=9), which were surgically resected between January 2014 and December 2015, at Kalinga Hospital Pvt. Limited, Bhubaneshwar, India. All the tumor samples were re-evaluated prior to selection for study, by two independent pathologists N.P. and A.M.K., and all the clinicopathological features were identified. The study was approved (BioEthics # MD-1) by institutional ethics committee, National Institute of Science Education and Research (NISER), Bhubaneswar, India. The tumor tissue was fixed in 10% buffered formalin, embedded in paraffin, and serially sectioned at 4-μm thickness. Sections were deparaffinized and rehydrated. Heat-induced epitope retrieval was done using microwave, in Envision target retrieval solution at high pH (Dako). To stop endogenous peroxidase activity, the specimens were immersed for 10 min, in Envision Peroxidase Blocker (Dako). The tissue section was treated with primary antibody for FRG1 (Biorbyt) at 1:100 dilution or for CD31 prediluted (Dako) followed by incubation for 1 h in a humidified chamber, at room temperature. Sections were washed and incubated in Envision Flex HRP secondary antibody (Dako). Liquid DAB substrate was used as the chromogen. The sections were then counterstained with Hematoxylin. The pathologists, blinded to patients’ background, scored the staining of FRG1 and microvessel density (MVD) count. HeLa cell block was stained as control, for FRG1 antibody.

The expression levels of FRG1 protein in the cytoplasm and nucleus of tumor cells were scored according to the intensity of staining and the percentage of positively stained cells. The surrounding uninvolved tissue served as a control for each sample. The staining ‘intensity’ measurements were classified into strong ‘3’, moderate ‘2’, weak ‘1’, or negative ‘0’ staining. The staining ‘percentage’ measurements in the tumor tissue were graded as ‘5’ if ≥67%, ‘4’ if 34–66%; ‘3’ if 11–33%, ‘2’ if 2–10%, ‘1’ if 1%, and ‘0’ if 0%, tumor cells were immunostained positive. Combining both factors, the data of FRG1 expression were defined by ‘Allred score’ [25], and further categorized into negative for 0–1, weak for 2–3, moderate for 4–6, and strong for 7–8. MVD was assessed as per Weidner et al. [26]. Briefly at first, the vascular hotspots were identified at 200× magnification and, microvessel counting was done for three hotspots at 400×. The hotspot with highest microvessel count was taken as the MVD count.

### Oncomine analysis

Oncomine cancer microarray database (http://www.oncomine.org) [27] was used to determine gene expression of FRG1 in various tumor types. For analysis, we set thresholds of *P*-value ≤0.05 and fold change ≥1.5. Thereafter, comparisons were drawn between tumor and normal group.

### Kaplan–Meier plotter analysis

Kaplan–Meier plotter analysis (http://kmplot.com/analysis) [28] was done to determine the prognostic value of *FRG1* gene expression. Overall survival (OS) parameter was chosen for the analysis in breast (*n*=4142), ovarian (*n*=1648), lung (*n*=2437), and gastric (*n*=1065) cancer. Patients were divided into two groups, with FRG1 high and FRG1 low, based on gene expression followed by comparative survival analysis between the two. Hazard ratio (HR) with 95% CI was calculated along with log rank *P*-value. *P*-value of ≤0.05 was considered to be significant.

### Statistical analysis

Statistical analysis was done using GraphPad Prism (version 7). For the expression analysis of FRG1 in the clinical specimen, Wilcoxon signed rank *t* test was performed in paired samples. Pearson correlation coefficient was derived to determine the association between tumor Allred score and MVD count. Student’s *t* test was used for cell-based assays to determine statistical significance between the experimental groups. *P*-value ≤0.05, was considered to be significant for all the tests.

## Results

### FRG1 levels affect endothelial cell function

**Figure 1:**
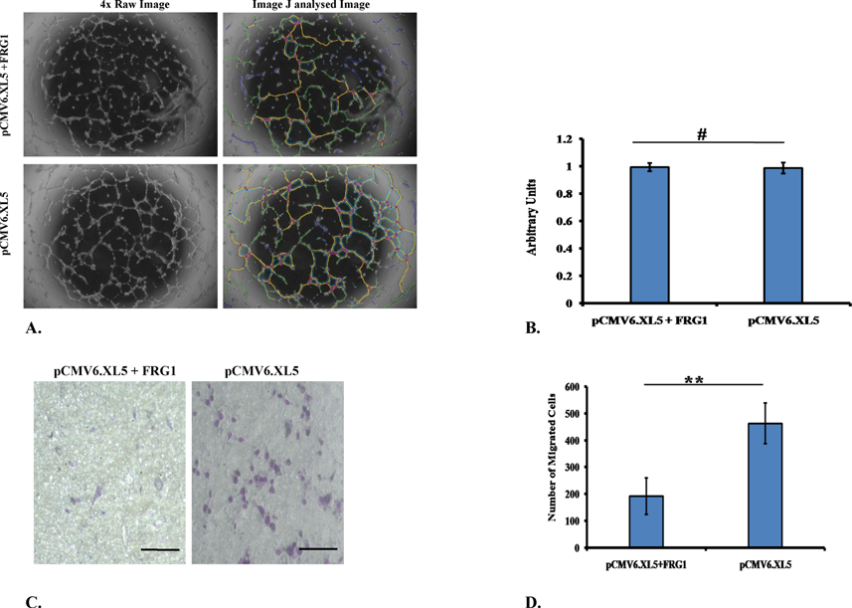
Effect of FRG1 expression on endothelial cell function (**A**) Representative image showing Matrigel tubule formation assay in HUVECs treated with conditioned medium, obtained from FRG1 expressing HEK293T cells and respective vector control; image shows reduced tubule formation in FRG1 overexpression set. (**B**) Graphical representation of cell proliferation assay data showing no significant change in proliferation of HUVECs when treated with conditioned medium obtained from HEK293T transfected with respective sets. (**C**) Representative images of HUVEC transwell migration assay in co-culture with HEK293T cells, transfected with FRG1 expression vector and empty vector control. (**D**) Graphical representation of HUVEC transwell migration assay showing significant reduction in HUVEC migration co-cultured with HEK293T expressing FRG1 (pCMV6.XL5.FRG1), compared with empty vector (pCMV6.XL5). # represents *P>0.05*, ** represents *P*<0.01.

To assess the role of FRG1 in angiogenesis, we used co-culture set up of endothelial cells (HUVECs) and epithelial cells (HEK293T). First, to identify the effect on endothelial cell differentiation, tubule formation assay was done. Treatment of HUVECs with conditioned medium, obtained from HEK293T cells transfected with FRG1 expression vector, led to reduced tubule formation compared with its control (Figure 1A). The analysis revealed that 13 out of 20 angiogenesis-related criteria, were significantly affected (*P*-value ≤0.05) (Table 1). Further, to identify the effect of FRG1 expression on endothelial cell proliferation, we performed cell proliferation assay on HUVECs. No effect on HUVEC cell proliferation was observed when treated with conditioned medium obtained from HEK293T cells transfected with FRG1 expression vector (Figure 1B).

Migratory properties of endothelial cells are essential for blood vessel development. Therefore, we checked the effect of FRG1 expression on HUVEC migration in the same co-culture setup where other assays were done. We observed a reduction in migration of HUVECs, there was statistically significant (*P*-value =0.009) difference between FRG1 overexpression set and empty vector control (Figure 1C,D).

### Altered FRG1 levels do not affect cell proliferation but it affects cell migration

**Figure 2:**
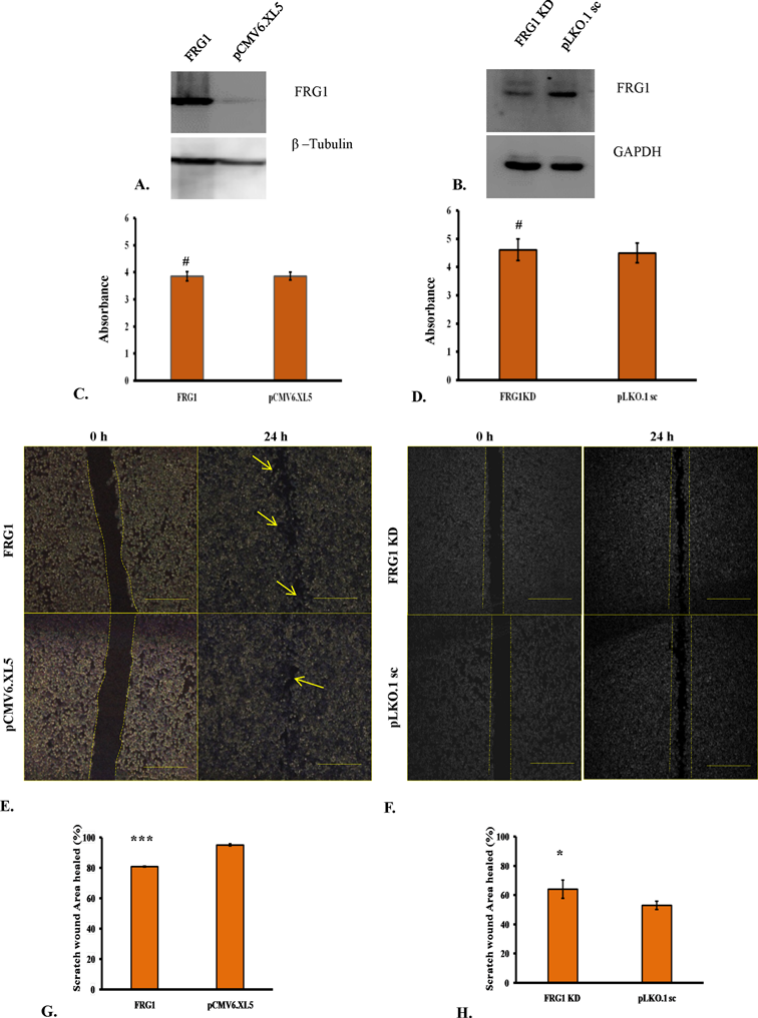
Effect of FRG1 expression on HEK293T cell proliferation and scratch wound healing (**A**) Shows Western blot to confirm ectopic expression of FRG1 in HEK293T. (**B**) Shows verification of reduced FRG1 levels after RNAi silencing in HEK293T, by Western blot. (**C**) Represents measurement of cell proliferation in HEK293T with ectopic expression of FRG1 compared with empty vector control (pCMV6.XL5), by MTS reagent. (**D**) Represents measurement of cell proliferation in HEK293T with knockdown of FRG1 compared with scrambled vector control (pLKO1.sc), by MTS reagent. (**E**) Shows representative images of scratch wound healing assay of HEK293T cells with ectopic expression of FRG1 and respective vector control (pCMV6.XL5). (**F**) Shows representative images of scratch wound healing assay of HEK293T with FRG1 knockdown and respective scrambled vector control (pLKO1.sc). (**G**) Represents representative graph for scratch wound healing assay of HEK293T cells with ectopic expression of FRG1, compared with empty vector control (pCMV6.XL5). (**H**) Shows representative graph for scratch wound healing assay of HEK293T cells with FRG1 knockdown, compared with empty vector control (pLKO1.sc). # represents *P>0.05*, * represents *P* <0.05, *** represents *P*<0.0001.

We chose HEK293T cells to test the oncogenic effects of FRG1, as these cells were not derived from cancer. Ectopic expression of FRG1 (Figure 2A) showed no change in cell proliferation compared with empty vector set (*P*-value =0.33) (Figure 2C). Similar results were observed when RNAi-based silencing reduced FRG1 levels (Figure 2B), no alteration in cell proliferation was observed compared with scrambled shRNA set (*P*-value =0.55) (Figure 2D).

**Figure 3:**
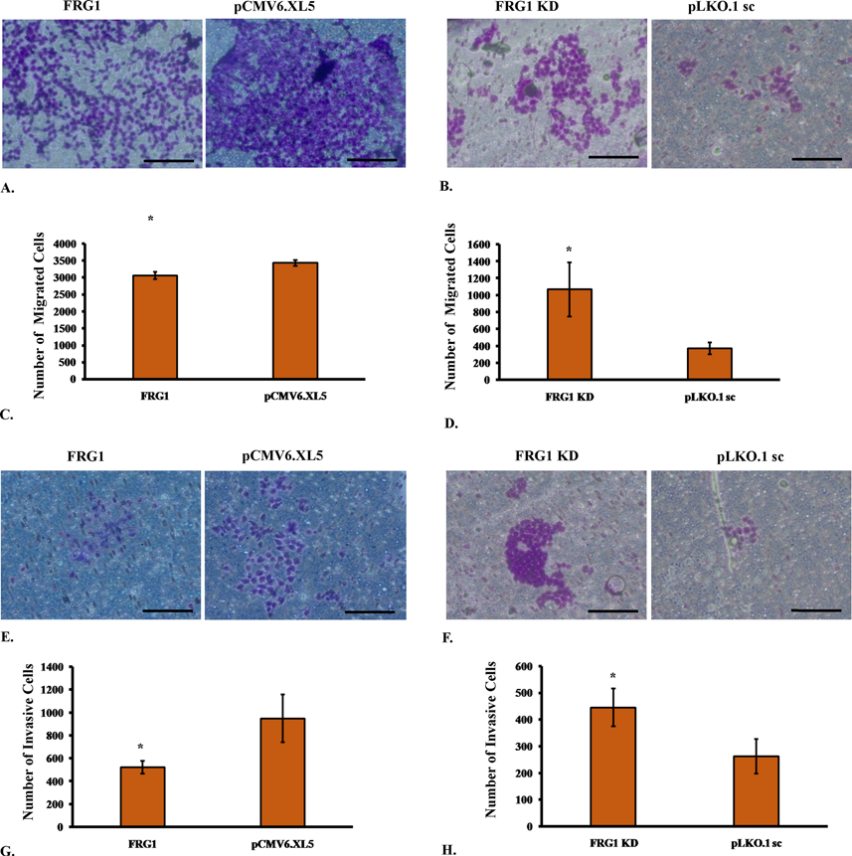
Effect of FRG1 expression on transwell migration and invasion (**A**) Shows representative images of transwell migration assay of HEK293T cells with ectopic expression of FRG1 and respective vector control (pCMV6.XL5). (**B**) Shows representative images of transwell migration assay of HEK293T with FRG1 knockdown and respective scrambled vector control (pLKO1.sc). (**C**) Represents representative graph for transwell migration assay of HEK293T cells with ectopic expression of FRG1, compared with empty vector control (pCMV6.XL5). (**D**) Shows representative graph for transwell migration assay of HEK293T cells with FRG1 knockdown, compared with empty vector control (pLKO1.sc). (**E**) Shows representative images of Matrigel invasion assay of HEK293T cells with ectopic expression of FRG1 and respective vector control (pCMV6.XL5). (**F**) Shows representative images of Matrigel invasion assay of HEK293T with FRG1 knockdown and respective scrambled vector control (pLKO1.sc). (**G**) Represents representative graph for Matrigel invasion assay of HEK293T cells with ectopic expression of FRG1, compared with empty vector control (pCMV6.XL5). (**H**) Shows representative graph for Matrigel invasion assay of HEK293T cells with FRG1 knockdown, compared with empty vector control (pLKO1.sc). * represents *P*<0.05.

Further, we wanted to evaluate the effect of FRG1 expression on cell migration. Effect on cell migration was assessed by scratch wound assay and transwell migration assay. In FRG1 overexpression set, healed wound area was found to be smaller than the empty vector control (Figure 2E). There was statistically significant (*P*-value <0.0001) difference in healed area between empty vector set (94.88 ± 0.66%) and FRG1 overexpression set (80.78 ± 0.35%) (Figure 2G). Similar trends were observed in transwell migration assay. Significantly (*P*-value =0.029) lesser number of cells migrated through the membrane in FRG1 overexpression set (3055 ± 110), compared with the control set (3425 ± 86) (Figure 3A,C).

To see whether reduction in FRG1 expression has opposite effect on migration, scratch wound healing assay was performed with HEK293T silenced for FRG1. As expected, the trends were observed to be just opposite to the overexpression set. Knockdown of FRG1 led to increased migration of cells, compared with scrambled control (Figure 2F). Statistically significant (*P*-value =0.036), more area (64 ± 6.28%) was healed in case of FRG1 knockdown compared with scrambled shRNA (53 ± 2.91%) (Figure 2H). Results of transwell migration assay supported the scratch wound healing assay data (Figure 3B). Number of cells (1069 ± 320) migrated in FRG1 knockdown set, was significantly (*P*-value =0.021) higher compared with cells (371 ± 71) migrated in scrambled shRNA set (Figure 3D). To sum up, all above assays advocate the role of FRG1 in cellular migration.

### FRG1 regulates cell invasion

During tumor progression, malignant cells invade through the extracellular matrix and metastasize into various organs. Thus, we performed Matrigel invasion assay to determine whether FRG1 expression affects invasiveness of the cell. Invasion data followed the trend of cellular migration (Figure 3E). Ectopic expression of FRG1 led to significant (*P*-value =0.026) reduction in cell invasion, with 520 ± 55 invasive cell count compared with 948 ± 208, of empty vector (Figure 3G). FRG1 knockdown led to significantly (*P*-value =0.029) enhanced cell invasion compared with scrambled shRNA (cell count: 445 ± 70 compared with 263 ± 65) (Figure 3 F,H). From these data, we can infer that FRG1 expression levels have a significant effect on invasiveness of HEK293T cells.

### FRG1 modulates cell migration and invasion by regulating *G-CSF* and *MMP10* expression

**Figure 4:**
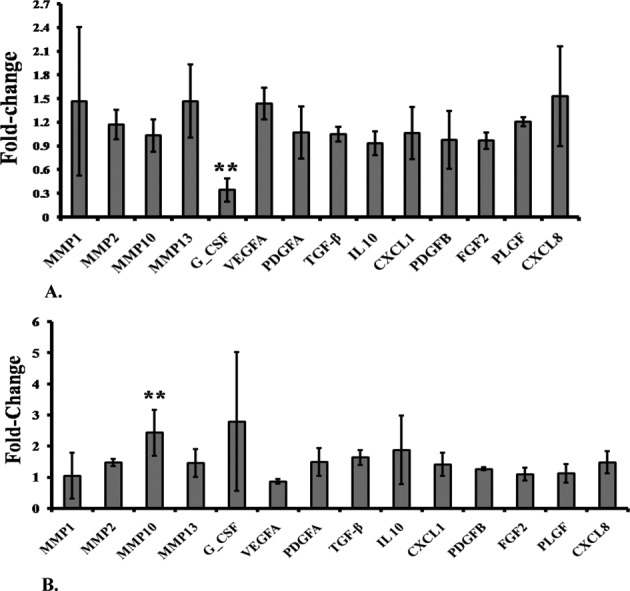
Effect of FRG1 expression on cell signaling molecules (**A**) qRT-PCR expression analysis shows effect of FRG1 expression on levels of various cytokines and MMPs, in HEK293T cells, transfected with FRG1 expression vector, compared with empty vector control. (**B**) Expression analysis of various cytokines and MMPs, in HEK293T cells with FRG1 knockdown, compared with scrambled vector control; X-axis shows the name of various signaling molecules and Y-axis shows the fold-change in expression of these molecules compared with their controls. ** represents *P*<0.01.

Further, we identified the signaling molecules affecting these cell properties through FRG1. Quantitative real-time PCR was performed for 7 MMPs and 11 tumor-associated cytokines. Only granulocyte colony stimulating factor (G-CSF) and matrix metalloproteinase 10 (MMP10) showed significant effect on their gene expression, in response to change in FRG1 expression. Expression analysis revealed that ectopic expression of FRG1 leads to reduction (3.3-fold, *P*-value =0.021) in G-CSF expression, which is the key molecule associated with cell migration and tumor progression (Figure 4A). Knockdown of FRG1 showed no specific effect on G-CSF expression, but led to increased expression of MMP10 by 2.48-fold (*P*-value =0.013) (Figure 4B). MMPs are known to play important role in tumor metastasis. These findings support the possible role of FRG1 in cell migration and invasion and identified the downstream molecules which might mediate the effect on cellular migration and invasion.

### *In silico* analysis shows reduced FRG1 expression in tumors and poor prognosis

**Figure 5:**
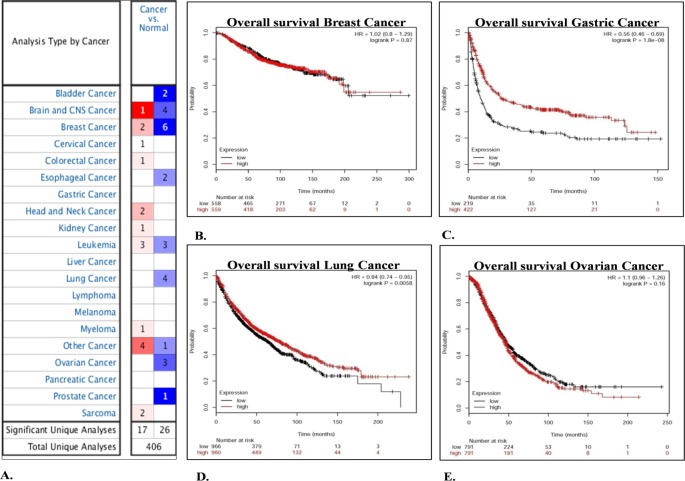
Determination of FRG1 expression levels and prognostic value by *in silico* analysis (**A**) Shows *FRG1* gene summary overview by Oncomine analysis. The view represents FRG1 expression in various tumor compared with normal datasets. The blue color represents down-regulation of FRG1 levels and red color represents up-regulation of FRG1 expression in a particular number of datasets. Intensity of color represents the level of up-regulation or down-regulation. (**B**–**E**) Represent Kaplan–Meier plotter analysis of (B) breast cancer, (C) gastric cancer, (D) lung cancer, and (E) ovarian cancer. Red line shows patients with expression above the median and black line shows patients with expression below the median. X-axis denotes the number of patients at risk with time in months and Y-axis denotes the probability of survival.

To look into FRG1 levels in tumor progression, *in silico* analysis was done. Oncomine analysis revealed that, out of total 462 analyses, *FRG1* expression was significantly affected in 43 analyses; 26 out of 43 analyses showed significant reduction in *FRG1* expression. On the contrary, 17 analyses showed significant up-regulation of *FRG1* (Figure 5A). Overall, *in silico* analysis of FRG1 expression showed that FRG1 expression is reduced in more oncomine datasets.

To further assert the importance of FRG1 expression, Kaplan–Meier plotter analysis was done for available cancer types, viz. breast, lung, gastric and ovarian (Figure 5 B,C,D,E). Survival analysis revealed that low *FRG1* expression was associated with poor prognosis in OS of patients, in lung cancer (HR =0.84, *P*-value =0.0058) (Figure 5 D) and in gastric cancer (HR =0.56, *P*-value =1.8 × 10^−8^) (Figure 5 C). Out of these four cancer types, Oncomine data suggest that *FRG1* expression was reduced in breast, lung, and ovarian cancer but in gastric cancer, not a single analysis was found to be significant.

### *In vivo* analysis shows reduced FRG1 protein expression in tumors

To further validate the *in silico* data and to derive correlation between FRG1 expression and tumorigenesis from cell-based studies, we checked FRG1 expression levels in tumor tissues. Additionally, we wanted to determine the general trend of FRG1 expression in tumors, so we took various cancer types and compared expression with its uninvolved counterpart. Surgically resected tumor tissue with uninvolved region of oral cancer, gastric cancer, and colon cancer were stained with FRG1 antibody. Significant reduction in FRG1 expression levels was observed in tumor, when compared with levels in uninvolved tissue.

**Figure 6:**
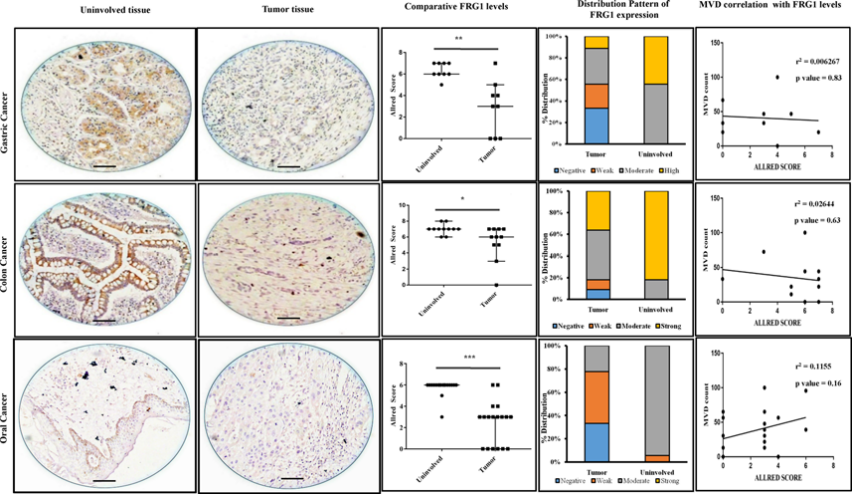
FRG1 levels and distribution pattern in tumor tissues and correlation with tumor MVD; top row represents gastric cancer, middle row represents colon cancer, and bottom row represents oral cavity cancer First two columns show representative images of FRG1 staining in uninvolved tissue and tumor tissue, respectively. The third column illustrates comparison of Allred score for FRG1 staining, between uninvolved tissue and tumor tissue. * represents *P*<0.05, ** represents *P*<0.01, *** represents *P*<0.0001. Distribution of staining pattern for the above-mentioned tumor types is represented by the fourth column. Last column represents correlation analysis between tumor, FRG1 staining Allred score, and MVD count.

In oral cancer, we observed that FRG1 levels were reduced in 61.11% of tumor cases, i.e. in 11/18 cases, compared with uninvolved region. FRG1 levels were mostly (90% cases) moderate in terms of staining in uninvolved region. On the other hand, staining in tumor area was weak to negative, in 80% cases (Figure 6). Comparison of Allred score for FRG1 in tumor and uninvolved tissue, showed significant (*P*-value =0.0001) reduction in FRG1 levels in tumor (median value =3) to uninvolved (median value =6) (Figure 6).

Similarly, in gastric and colon cancers, FRG1 levels in tumor were reduced in 66.66% (6/9) and 63.63% (7/11) compared with the uninvolved control, respectively. Distribution pattern of FRG1 staining in gastric cancer revealed that more than 40% cases had strong staining and 60% cases had moderate staining in uninvolved tissue, whereas in tumor total 40% cases belonged to strong to moderate group (Figure 6). Comparison of Allred score showed significant (*P*-value =0.0078) reduction in tumor (median =3), compared with uninvolved (median =6) (Figure 6). In colon cancer, distribution pattern showed that ~80% cases had strong FRG1 staining in uninvolved tissue, compared with ~40% cases with strong FRG1 staining in tumor tissue (Figure 6). Allred score analysis indicated significant (*P*-value =0.0195) reduction in Allred score for tumor (median =5), compared with uninvolved tissue (median =7) (Figure 6). Overall, these data indicate a possible role of FRG1 in tumor progression.

### FRG1 levels are not associated with tumor angiogenesis

Since earlier studies suggest association of FRG1 with vascular abnormalities and our *in vitro* data show that higher levels of FRG1 lead to reduction in tubule formation, therefore, we checked whether FRG1 level is associated with tumor angiogenesis. For identification, we performed a correlation analysis between Allred score of tumors with MVD count of the respective tumor type. The correlation coefficient for MVD compared with Allred score of oral, gastric, and colon cancers were r^2^ =0.115 (*P*-value =0.16); r^2^ =0.026 (*P*-value =0.63) and r^2^ =0.006 (*P*-value =0.83), respectively (Figure 6). Generally, correlation analysis suggests that FRG1 expression levels may not have a role in tumor angiogenesis.

## Discussion

FRG1 has been very well implicated in FSHD, but in recent times studies have reported functional insights into various cellular and physiological processes [7,12,13]. Based on the indirect indications, we decided to investigate the role of FRG1 in angiogenesis and tumorigenesis. The first-time study reports the effect of FRG1 expression on cellular properties of primary endothelial cell and epithelial origin cell line. The role of FRG1 in angiogenesis remains questionable. The first idea of involvement of FRG1 in angiogenesis regulation was formed by a simple observation, that is, 75% of FSHD patients were diagnosed with retinal vasculature abnormalities [8–10]. To elucidate involvement of FRG1 in angiogenesis, FRG1 transcription signatures of FSHD patients (*n* =19) were compared with healthy individuals (*n* =30). The findings of the study showed unaltered FRG1 levels in patients with vasculature abnormalities [9]. On the contrary, developmental studies on *Xenopus* showed that frg1 is crucial for angiogenesis but not for vasculogenesis [7]. Our *in vitro* data suggest involvement of FRG1 in angiogenesis, as we observed FRG1 overexpression led to reduction in tubule formation and migration of endothelial cells. Further, we could not observe association between FRG1 levels in tumor and tumor MVD. The underlying reason could be the small sample size used for the association. Moreover, we did not have enough number of tumors from different stages and grades. It is possible that FRG1 expression correlates with angiogenesis only in initial stage of tumorigenesis. Study in better sample size, where stratification is possible, can help us ascertain the role of FRG1 in angiogenesis. Hence *in vitro* data provide us with the first-hand information regarding involvement of FRG1 in angiogenesis in humans; but question still remains for the tumor patients which provides parallel insights into the findings in FSHD patients with vasculature abnormalities [9].

Our data suggest that FRG1 may have tumor suppressor activity as its overexpression inhibits cellular migration and invasion and vice versa, and both these properties are important for the metastatic growth of tumor. Reduced expression of FRG1 in patient tumor tissues also supports tumor suppressive role of FRG1. Further evidence can be drawn from regulation of angiogenesis by FRG1. Reduced angiogenesis during FRG1 overexpression clearly points toward tumor suppressive role of FRG1. Developmental studies in *Xenopus* reported that FRG1 overexpression led to increased delamination and migration of muscle cells from myotome. The present study concluded that FRG1 may be essential for epithelial to mesenchymal transition (EMT) or mesenchymal to epithelial transition (MET) [3]. Our findings suggest other way, as we observed reduction in cellular migration and invasion, on FRG1 overexpression and vice versa. Another study supports our data indirectly, where FRG1 expression is reduced in migratory breast cancer cells [29]. FRG1 overexpression led to atrophy in muscle cells and reduced cell proliferation of C2C12 myoblasts, which was restored over time with reduction in FRG1 expression [6]. Nevertheless, an important question still remains to be answered, i.e. whether FRG1 expression affects cell proliferation during tumor progression. In the present study, FRG1 levels did not affect cell proliferation of HEK293T cell line. To further characterize the involvement of FRG1 in tumorigenesis, animal model based studies are required. The statement is noteworthy as mouse model with FRG1 overexpression is available but no animal models are known for FRG1 knockout/knock down, which would be appropriate model to provide insights into tumorigenesis.

To find the insights into molecular mechanism of FRG1-mediated tumorigenesis, we checked expression of various signaling molecules. We found that ectopic expression of FRG1 leads to reduction in expression of G-CSF, a hematopoietic growth factor that induces proliferation and differentiation of hematopoietic stem cells of neutrophils [30–33]. Studies have established the role of G-CSF in tumor progression. Administration of G-CSF leads to increase in protumorigenic factors, VEGF, and TGF β [34–36]. Also, G-CSF enhances migration and proliferation of gastric and colon carcinoma cells which is further supported by findings in head and neck cancer [37–39]. Reduction in G-SCF levels could be primary reason for reduction in cellular migration and invasion of HEK293T cells, on ectopic expression of FRG1. G-CSF levels might be critical for our *in vitro* finding regarding endothelial cell function. G-CSF levels are known to promote angiogenesis and tumorigenesis [40]. Direct mediation of G-CSF facilitates angiogenesis and reduces ischemia, as observed in ischemic model system [41]. Therefore, the effects observed in our *in vitro* study could be attributed to reduced G-CSF expression with FRG1 overexpression in HEK293T.

Knockdown of FRG1 in HEK293T showed increased expression of MMP10. MMPs are a family of zinc-related endoproteases involved in tumor invasion, metastasis, and angiogenesis [42]. MMP10 belongs to stromelysin subfamily of MMPs. Higher MMP10 expression has been reported in lung cancer [43,44], head and neck cancer [45,46], oral cavity cancer, esophagus cancer [47], and cervical tumors [48]. Higher expression of MMP10 directly modulates cell migration and enhances invasiveness of the cell [49]. Above-mentioned studies support that up-regulation of MMP10 on FRG1 silencing, might be responsible for enhanced cell migration and invasiveness.

Since G-CSF and MMP10, which are known to promote tumor growth, levels were affected by FRG1, we looked for FRG1 expression in tumor tissues. Our first study is to identify reduced expression of FRG1 in tumor tissues. Expression of FRG1 was higher in uninvolved epithelial tissues, compared with the tumor. Previous study by Hanel et al. [50] showed positive staining of FRG1 in skin epithelial lining and sweat glands. The staining patterns were consistent in our study, as basal layer of stratified epithelium, sweat glands, and sebaceous glands were positively stained for FRG1. We observed that FRG1 is predominantly localized in cytoplasm but nuclear positivity was also observed in some cases, as reported previously [11,13,50]. Further, change in cellular migration and invasion properties with altered FRG1 levels, supported its role in tumorigenesis. One aspect which suggests that FRG1 can be multifunctional is localization. FRG1 is known to be associated with spliceosome complex and, have role in mRNA transport and RNA splicing [12,13]. On the other hand, it is associated with actin bundling protein, providing structural integrity to the cell [13]. The localization of FRG1 is predominantly nuclear in cell lines such as mDEC6 and C2C12 [14,50]. Whereas it is predominantly cytoplasmic in, human skeletal muscle myoblasts (HSMMs), and muscle derived stem cells (MDSCs) [50]. In mDEC6 cells, BMP4 treatment of leads to change in FRG1 localization from nucleus to cytoplasm [14]. Since localization of FRG1 is critical to its function, with above-mentioned studies, we can get a clear idea that FRG1 functionality may vary amongst different systems and it depends on the activity of stromal components, as observed in animal models.

In summary, the present study provides first insights into role of FRG1 in tumorigenesis. *In silico, in vivo*, and *in vitro* analysis of FRG1 levels revealed that loss of FRG1 expression promotes tumorigenesis; but its effect on angiogenesis needs a thorough understanding [7]. Effect of FRG1 expression on cellular migration and invasion might be through G-CSF and MMP10, which might have also dictated the *in vitro* endothelial cell function. However, the specific molecular mechanism of FRG1 is yet to be understood.

## Conclusion

FRG1 expression is reduced in tumor tissues. FRG1 may affect tumor progression by affecting tumor cell migration and invasion. FRG1 regulates G-CSF and MMP10 expression which affects tumorigenesis and angiogenesis. However, identification of detailed molecular mechanism associated with FRG1 in suppression of cellular migration, invasion, and angiogenesis, needs further study.

## Supporting information

**Supplementary Table 1:** Genes with list of primers, for which expression was determined in HEK293T cells with altered FRG1 expression

## Abbreviations

95% CI: 95% confidence interval
BMP4: bone morphogenetic protein 4
DMEM: Dulbecco’s modified Eagle’s medium
FRG1: FSHD region gene 1
FSHD: facioscapulohumeral muscular dystrophy
G-CSF: granulocyte-colony stimulating factor
GAPDH: glyceraldehyde 3-phosphate dehydrogenase
HEK293T: human embyonic kidney
HR: hazard ratio
HUVEC: human umbilical vein endothelial cell
MMP: matrix metalloproteinase
MMP10: matrix metalloproteinase 10
MVD: microvessel density
OS: overall survival
qRT-PCR: quantitative real time PCR
TGF β: transforming growth factor β
VEGF: vascular endothelial growth factor
