# Supplementary material for: Increased FSHD region gene1 expression reduces in vitro cell migration, invasion, and angiogenesis, ex vivo supported by reduced expression in tumors
Source: Biosci Rep. 2017 Oct 27;37(5):BSR20171062. doi: 10.1042/BSR20171062 (PMC5665614; doi:10.1042/BSR20171062)
Supplement: Supplementary file 1 [file BSR20171062_Supp1.pdf]

Supplementary Table 1: Genes with list of primers, for which expression was determined in HEK293T cells with altered FRG1 expression

Gene	Primer 5`---- 3`
MMP1 F	AGAGCAGATGTGGACCATGC
MMP1 R	TTGTCCCGATGATCTCCCCT
MMP2 F	CGTCGCCCATCATCAAGTTC
MMP2 R	CAGGTATTGCACTGCCAACTC
MMP3 F	CACTCACAGACCTGACTCGG
MMP3 R	AGTCAGGGGGAGGTCCATAG
MMP8 F	AAGCCAGGAGGGGTAGAGTT
MMP8 R	TTTTCCAGGTAGTCCTGAACAGT
MMP9 F	TTCAGGGAGACGCCCATTTC
MMP9 R	AACCGAGTTGGAACCACGAC
MMP10 F	AGTTTGGCTCATGCCTACCC
MMP10 R	TTGGTGCCTGATGCATCTTCT
MMP13 F	GTTTGCAGAGCGCTACCTGA
MMP13 R	GACTGCATTTCTCGGAGCCT
FGF2F	GCTGTACTGCAAAAACGGGG
FGF 2 R	TAGCTTGATGTGAGGGTCGC
PLGF F	CCATGCAGCTCCTAAAGATCC
PLGF R	TCCTCCTTTCCGGCTTCA
CXCL1 F	AACCGAAGTCATAGCCACAC
CXCL1 R	GTTGGATTTGTCACTGTTCAGC
CXCL8 F	ACCGGAAGGAACCATCTCAC
CXCL8 R	GGCAAAACTGCACCTTCACAC
IL 10 F	AAGACCCAGACATCAAGGCG
IL 10 R	AATCGATGACAGCGCCGTAG
PDGFA F	GCCAACCAGATGTGAGGTGA
PDGFA R	GGAGGAGAACAAAGACCGCA
PDGFB F	ACCTGCGTCTGGTCAGC
PDGFB R	ATCTTCCTCTCCGGGGTCTC
GM-CSF F	CTGGAGCTGTACAAGCAGGG
GM-CSF R	ACAGGAAGTTTCCGGGGTTG
G-CSF F	AGCAAGTGAGGAAGATCCAGG
G-CSF R	TTGTAGGTGGCACACTCACTC
VEGFA-F	ATCTGCATGGTGATGTTGGA
VEGFA-R	GGGCAGAATCATCACGAAGT
TGF-beta-F	GCAACAATTCCTGGCGATACC
TGF-beta-R	AAAGCCTCAATTTCCCCTCC
